# Draft Genome Sequence of Blautia sp. Strain BCRC 81119, Isolated from Human Feces

**DOI:** 10.1128/MRA.00812-18

**Published:** 2018-08-02

**Authors:** Chien-Hsun Huang, Jong-Shian Liou, Chun-Lin Wang, Lina Huang

**Affiliations:** a Bioresource Collection and Research Center, Food Industry Research and Development Institute, Hsinchu, Taiwan, Republic of China; Louisiana State University

## Abstract

Blautia sp. strain BCRC 81119 was isolated from a fecal sample from a 34-year-old male in Taiwan. The genome assembly comprised 4,098,441 bp, with a 43.95% G+C content.

## Abstract (precis)

Blautia sp. strain BCRC 81119 was isolated from a fecal sample from a 34-year-old male in Taiwan.

## ANNOUNCEMENT

Culturomics technology is a powerful tool for discovering new species in the human gut, and it can be used to complement metagenomics by overcoming the depth bias issue (1). During a study aimed at isolating novel bacterial species present in the human gut, we isolated strain BCRC 81119 from a fecal sample of a 34-year-old male in Taiwan by using a culturomics approach (2, 3). Based on the sequencing of the complete 16S rRNA gene, BCRC 81119 was found to exhibit 99.51% sequence identity with Blautia massiliensis DSM 101187 (GenBank accession number LN913006). In addition, the average nucleotide identity (ANI) and digital DNA-DNA hybridization (dDDH) values between BCRC 81119 and DSM 101187 were 98.07% and 86.2%, respectively. These values were clearly higher than the generally accepted cutoff threshold of 95 to 96% and 70%, respectively, for delineation of prokaryotic species, thus confirming that strain BCRC 81119 belongs to the species B. massiliensis. It has been reported that B. massiliensis might be an important species for human health, because it has been frequently isolated and detected in the human gut (4).

Blautia sp. strain BCRC 81119 is a rod-shaped, strictly anaerobic, non-spore-forming bacterium. It grows on yeast extract, Casitone, fatty acid, and glucose (YCFAG) medium (5) within 48 h at 37°C under anaerobic conditions. Genomic DNA was extracted using an EasyPrep HY genomic DNA extraction kit (Tools, Taiwan, Republic of China) following the manufacturer’s protocol. Libraries were constructed using approximately 1 μl of genomic DNA, and the draft genome was sequenced from an Illumina paired-end library with an average insert size of 350 bp by using an Illumina HiSeq 4000 instrument with a paired-end 125-bp read length at the Beijing Novogene Bioinformatics Technology Co., Ltd. (Beijing, People's Republic of China). The low-quality sequences of the paired-end and mate pair reads were analyzed using an in-house program by Novogene Bioinformatics Technology Co., Ltd. Sequencing of strain BCRC 81119 generated 1,216 Mb of data. After adapter filtering and quality trimming, *de novo* assembly of the reads was performed using SOAP*denovo* (6), which resulted in 77 scaffolds with an *N*_50_ value of 112,070 bp. The draft genome contains 4,098,441 bp, with a G+C content of 43.95%, and was annotated using the NCBI Prokaryotic Genomes Automatic Annotation Pipeline (PGAAP) to obtain 3,922 predicted genes, 14 rRNAs, 87 tRNAs, and 4 noncoding RNAs (7). Annotation of the protein-coding genes with Rapid Annotations using Subsystems Technology (RAST) (8) predicted 4,429 protein-coding genes, 2,306 of which had functional categories of SEED subsystems, while analysis with antiSMASH version 4.1 (9) predicted one sactipeptide biosynthetic gene cluster.

### Data availability.

This whole-genome shotgun project has been deposited at DDBJ/ENA/GenBank under the accession number QJHD00000000 (BioProject number PRJNA473893). The version described in this paper is the first version.
